# High-Throughput Nanofabrication of Infra-red and Chiral Metamaterials using Nanospherical-Lens Lithography

**DOI:** 10.1038/srep03339

**Published:** 2013-11-28

**Authors:** Yun-Chorng Chang, Sih-Chen Lu, Hsin-Chan Chung, Shih-Ming Wang, Tzung-Da Tsai, Tzung-Fang Guo

**Affiliations:** 1 Department of Photonics and Advanced Optoelectronic Technology Center, National Cheng Kung University, Tainan 701, Taiwan

## Abstract

Various infra-red and planar chiral metamaterials were fabricated using the modified Nanospherical-Lens Lithography. By replacing the light source with a hand-held ultraviolet lamp, its asymmetric light emission pattern produces the elliptical-shaped photoresist holes after passing through the spheres. The long axis of the ellipse is parallel to the lamp direction. The fabricated ellipse arrays exhibit localized surface plasmon resonance in mid-infra-red and are ideal platforms for surface enhanced infra-red absorption (SEIRA). We also demonstrate a way to design and fabricate complicated patterns by tuning parameters in each exposure step. This method is both high-throughput and low-cost, which is a powerful tool for future infra-red metamaterials applications.

Metamaterials (MMs) are artificial materials which consist of sub-wavelength elements and their spatially averaged response can be recognized as a homogeneous medium with a characteristic effective permittivity (ε) and permeability (μ)^1^,^2^. These man-made materials demonstrate optical properties that are not found in nature and make possible for some exotic applications, such as invisible cloak^3^ and perfect lenses^4^. Recently, visible MMs have been the focus for researches worldwide^5^,^6^,^7^, but the progress was severely limited due to the difficulty in nanofabrication. In contrast, infra-red (IR) MMs are much easier to be fabricated and have been leading to novel applications, such as perfect absorbers (PAs)^8^,^9^,^10^,^11^,^12^ and electromagnetically induced transparency (EIT)^13^,^14^. These IR MMs can be used as high efficient thermal absorbers/emitters for thermophotovoltiac cells^15^ or sensitive refractive index sensors for biosensors^11^,^14^,^16^. Meanwhile, asymmetric nano-cross arrays have also been reported to work as quarter wave monopole antenna elements which are compatible with chip-based fabrication techniques^17^. In recent years, a new class of metamaterials, known as planar chiral metamaterials (PCMs), has invoked much interest in the scientific community due to their large optical activities and polarization conversion can be achieved in a PCM structure with a thickness at only a fraction of a wavelength^18^,^19^,^20^,^21^.

When illuminated the metal nanoparticles with resonant light that matches their localized surface plasmon resonance (LSPR), strong electric field occurs near the surface and is useful to boost the Raman scattering signal for ultrasensitive surface-enhanced Raman scattering (SERS) measurements^22^ and the absorption signal from the vibrational modes of molecules for surface-enhanced IR absorption (SEIRA) spectroscopy. Both techniques can reveal the vibrational fingerprints of bio-molecules. However, the sensitivity is severely limited due to the small intrinsic absorption cross-sections. SEIRA is usually performed with chemically prepared or roughened metal surfaces^23^,^24^ and its enhancement factor is limited to 10–100 because of the random distributions of the surface roughness. Recently, SEIRA has been demonstrated from engineered metal nanostructures, such as nano-shells^25^, nano-crescents^26^, and nano-bars^27^,^28^. These nanostructures exhibit uniform size distributions and can be arranged periodically on top of a surface. These periodic nanostructures can easily achieve a SEIRA signal enhancement of 10^4^, which is capable to detect the vibrational signals from a single protein monolayer.

LSPR of an individual nanoparticle can also be tuned by varying the size and shape of the nanoparticle^29^,^30^,^31^,^32^,^33^. Nanoparticles with more complex shapes, such as nano-crescents^34^ and nano-crosses^35^,^36^, have also been investigated for various LSPR applications. These complex nanostructures can be precisely fabricated using focused-ion-beam milling (FIB) or electron-beam lithography (EBL). However, the low throughput of these techniques is not ideal to fabricate samples with sizes as large as 1 cm^2^ that is typical for IR absorption spectroscopy. Nanofabrication methods with high-throughput capability, such as interference lithography^37^,^38^,^39^,^40^, hole-mask colloidal lithography^41^ and nano-imprint lithography^42^, are the preferred nanofabrication techniques. Furthermore, fabrication of nanoscale triangle arrays have been reported using Nano-sphere Lithography^43^. Micro-spheres have been reported as focusing lenses to focus the incident laser light and ablate the underlining materials^44^,^45^,^46^,^47^. These spheres can also be used as phase-shifting lithography masks to fabricate two-dimensional nanostructures^31^,^33^,^48^,^49^. In our previous reports, large-scale nanodisk arrays are successfully fabricated using this method, which we referred it as “Nanospherical-Lens Lithography (NLL)”^50^,^51^. Furthermore, the micro-/nano- spheres were also reported to be used for micro-/nano- fabrication and imaging^52^,^53^.

Here we demonstrate the fabrication of IR MMs and PCMs using NLL simply by replacing the light source with a hand-held ultraviolet lamp. The fabricated nano-ellipse arrays are perfect platforms for SEIRA. More complicated IR MMs and PCMs are also demonstrated by precisely controlling the multiple exposure sequences. The proposed method is possible for large-scale fabrication with very low fabrication cost, which should be beneficial for future industrialization of IR MMs and PCMs.

## Results

### Nano-ellipse arrays fabricated by replacing the light source of Nanospherical-Lens Lithography with a hand-held UV lamp

**Figure 1:**
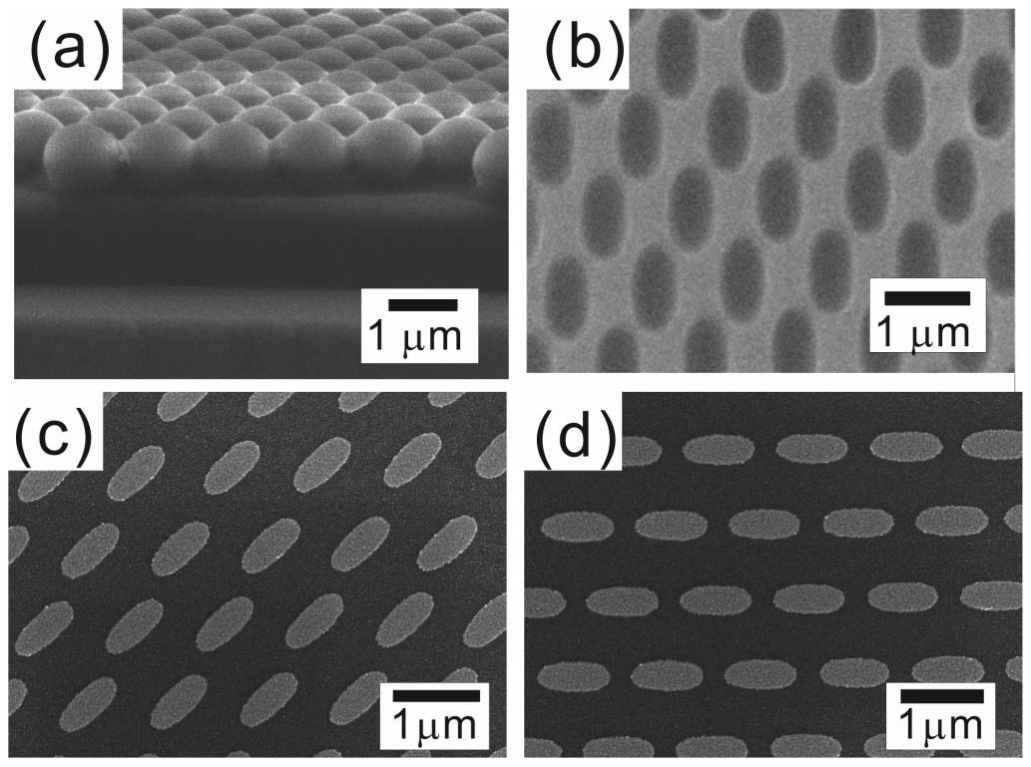
SEM images of (a) the nanosphere array on top of photoresist, (b) the elliptical photoresist hole arrays. (c) and (d) the fabricated nano-ellipse arrays with the long-axis pointing to two different directions.

The first step for the NLL is to align a single-layered nanosphere array on top of a photoresist (PR) thin film, as illustrated in the scanning electron microscope (SEM) image in Fig. 1(a). The subsequent UV exposure is usually done with a commercial UV aligner as the light source and round-shaped PR hole patterns reveal after PR development^50^. Although commercial UV aligners are standard instruments in semiconductor factories, they are actually quite expensive. Therefore, we have tried to replace it with a hand-held UV lamp, which is cheaper and easily available. To our surprise, elliptical PR hole arrays reveal after PR develop as shown in Fig. 1(b). The diameter of spheres is 1 μm. By controlling the orientation of the lamp, nano-ellipse arrays with different configurations can be fabricated, as shown in Figs. 1(c) and (d). The long axis of the fabricated nano-ellipse is always parallel to the lamp direction.

**Figure 2:**
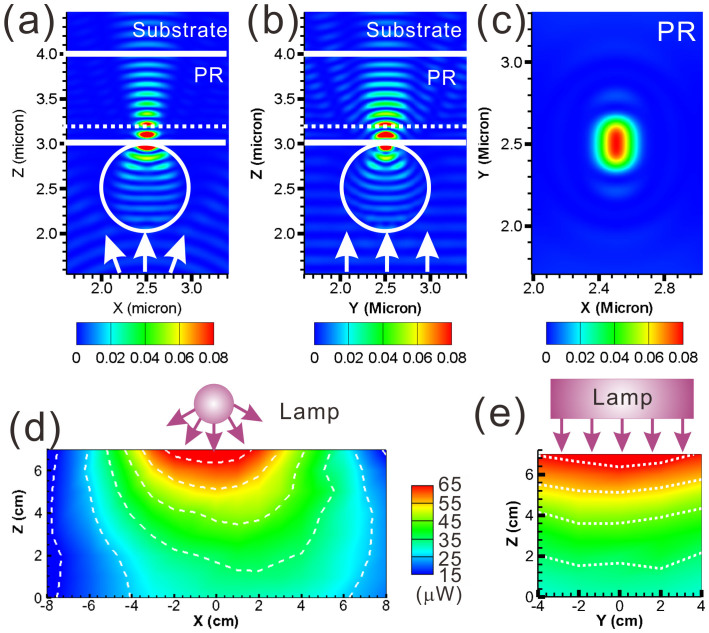
Simulated field energy distributions across the (a) xz-plane and (b) yz-plane that demonstrates two different focusing behaviors of the incident light when using UV lamp as the light source. The field energy distribution across the xy-plane (z = 3.2 μm) is shown in (c). The dash lines in (a) and (b) indicate the location of z = 3.2 μm plane. Measured light intensity distribution of the UV lamp across the (d) xz-plane and (b) yz-plane. The UV lamp is positioned at z = 15 cm and orientated along the y-axis.

In order to explain why elliptical patterns forms when the UV lamp is used as the light source, the light intensity distribution of the UV lamp is measured and shown in Figs. 2(d) and (e). UV lamp is positioned at a height of z = 15 cm and oriented along the y-axis. The light intensity is recorded every 1 cm along all three axes. It is very clear that the light distribution across the xz-plane shown in Fig. 2(d) is very similar to a regular point source while the distribution across the yz-plane shown in Fig. 2(e) is an uniform light source. The results are consistent with the fact that the back reflector exists in a UV lamp and the light is coming out of it with two different propagating behaviors. In order to further illustrate why these elliptical PR holes form, theoretical simulations were performed using a three-dimensional finite-difference time-domain (3D-FDTD) method. Figs. 2(a)–(c) illustrate the simulated field energy distributions across the xz-, yz-, and xy-planes near a nanosphere at a diameter of 1 μm. The UV light (λ = 365 nm) is emitting from a line-shaped source that is parallel to the y-axis. The propagation of the incident light across the xz- and yz-planes demonstrates different behaviors, which are clearly visible in Figs. 2(a) and (b). The difference in the propagation behavior between the xz- and yz-planes results in different focusing spot sizes between two different polarizations. Fig. 2(c) illustrates the simulated field energy distribution across the z = 3.2 μm plane, which are also marked as white dash lines in Figures 2(a) and 2(b). The field energy distribution is an elliptical-shaped pattern, which explains why elliptical PR holes are formed.

### Tunable size and aspect ratio of nano-ellipses

**Figure 3:**
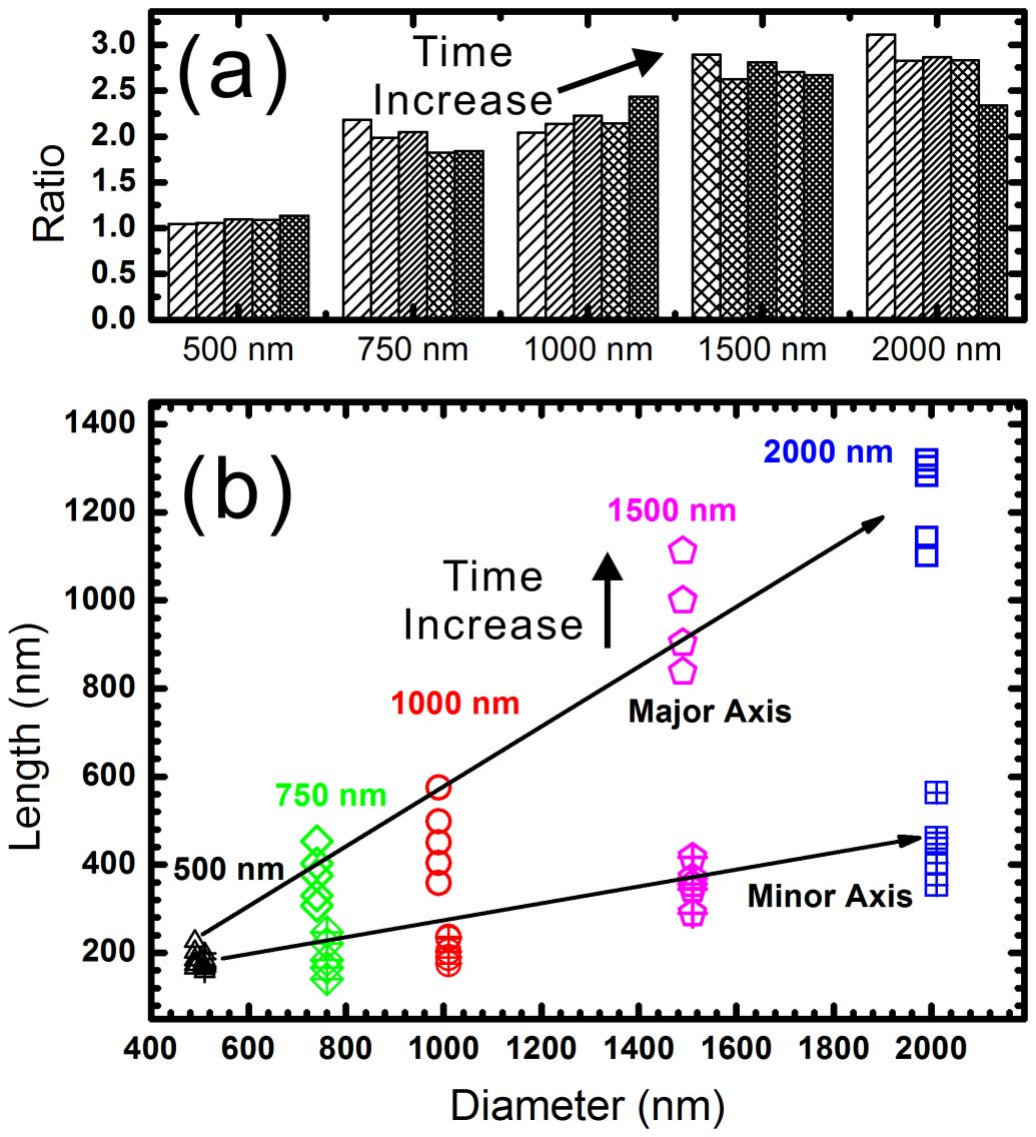
(a) Aspect ratio and (b) major and minor axis lengths of the fabricated nano-ellipses using nanospheres at various diameters and different exposure time.

The fabricated nano-ellipses using spheres at a diameter of 1 μm are too large to exhibit a surface plasmon resonance in the visible light region, smaller nano-ellipses with smaller periodicity are necessary. However, it surprised us that only round PR holes were fabricated when the nanospheres at a diameter of 500 nm are used. Therefore, the aspect ratio between the lengths of major and minor axes strongly depends on the diameter of the nanospheres. Micro/Nano-spheres with different diameters are subsequently investigated and we have discovered that the aspect ratio is strongly affected by the diameters of the spheres. As illustrated in Fig. 3(a), the aspect ratio increases to around 3 when the diameter of the sphere increases to 2 μm. 5 different exposure durations are investigated for each size and the corresponding ratio is illustrated as a bar in Fig. 3(a). For each size of sphere, the column located at the right represents a longer exposure time. Figs. 3(b) and (c) represent the lengths of the major and minor axes of the fabricated nano-ellipses using spheres with different diameters. For each size of sphere, the data point at higher location represents a longer exposure duration time. In these figures, we have demonstrated that the length and the aspect ratio of the fabricated nano-ellipses can be tuned by controlling the exposure time and the diameters of spheres. The ratio is tunable between 1 and 3 and the length of the major axis is tunable between 200 nm and 1.4 μm.

### Nano-ellipse array as ideal platforms for SEIRA

**Figure 4:**
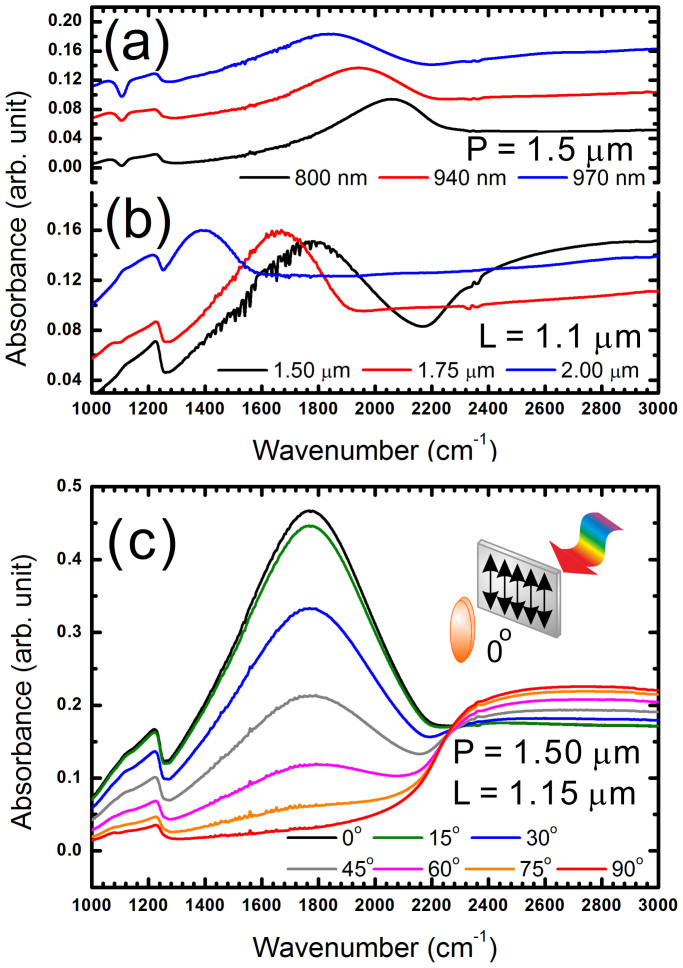
FTIR spectra of Au nanoellipse arrays with varying (a) length for long axis and (b) periodicity. (c) Polarization-dependent FTIR spectra of nanoellipse arrays which long-axis length is 1.15 mm and periodicity is 1.5 mm. Inset in (c) illustrates that 0° corresponds to the polarizer is aligned along the long-axis of the nanoellipses.

One of the major applications for the fabricated nano-ellipse arrays are to be used as platforms for SEIRA. By fabricating the nano-ellipses with their long axis around 1.2 μm, the LSPR of the major axis is between 1000 cm^−1^ to 2000 cm^−1^. This spectral range covers most of the vibrational energy spectra of many chemical and biological species. SEIRA within this spectra range exhibits high potential for ultra-sensitive biosensing capabilities. Fig. 4(a) illustrates the LSPR of the nano-ellipse arrays with different lengths of major axis. The periodicity is 1.5 μm. The LSPR shifts to a lower energy when the length of the major axis becomes smaller. The LSPR peaks are also affected by the periodicity, which is located near 2200 cm^−1^. In Fig. 4(b), we fix the length of the major axis at 1.1 μm and vary the periodicity. A shift to lower energy is observed when using a larger periodicity. A nano-ellipse should exhibit a polarization-dependent optical property. Fig. 4(c) illustrates the polarization-dependent extinction spectra of the fabricated Au nano-ellipse arrays. The periodicity and the length of the major axis are 1.5 and 1.15 μm, respectively. The polarization-dependent absorbance of the fabricated arrays further verifies the observed extinction response originates from the LSPR of the nano-ellipse arrays. Therefore, we have demonstrated the ability to perform precise tuning of the nano-ellipse arrays for a specific infrared band. By using the polarization-dependent FTIR measurement, we can maximize the FTIR signal to improve the signal intensity of SEIRA.

### Complex IR and chiral metamaterials fabricated with different multiple exposures

**Figure 5:**
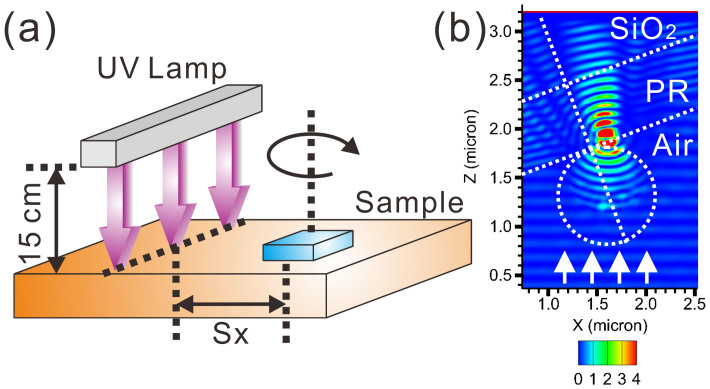
(a) Schematical illustration of multiple exposures. The vertical distance between UV lamp and the sample is kept at 15 cm. The sample can be rotated and shifted along one direction. Each exposure is denoted by 3 parameters. E_X_(S_x_, Angle, Duration). “X” denotes the exposure number, “S_x_” the shifting distance, “Angle” the angle between the sample and the lamp, and “Duration” the exposure duration. Multiple exposures after different rotating angles at different S_x_ can produce different types of metamaterials. (b) Simulated field energy distributions across the xz-plane when exposure the samples at a shifted location. The light is focused at an offset center location.

In addition to the nano-ellipse arrays, the proposed method can be used to fabricate more complex-shaped metamaterials after multiple exposures. Fig. 5(a) illustrates the experimental configuration for the multiple exposures. The UV lamp is positioned at a constant height of 15 cm above the sample. The sample can be rotated or shifted along the direction perpendicular to the lamp direction, where S_x_ denotes the shifting distance. The sample shift causes the UV light to incident the surface at an angle. Fig. 5(b) illustrates the simulated field energy distribution across the xz-plane near a nanosphere at a diameter of 1 μm when the UV light (λ = 365 nm) is incident to the surface at an angle of 30°. The field energy distribution reveals that the incident light exposes the underneath photoresist with an offset center position, which is useful to fabricate complicated metamaterials when exposing with a line-shaped UV lamp.

**Figure 6:**
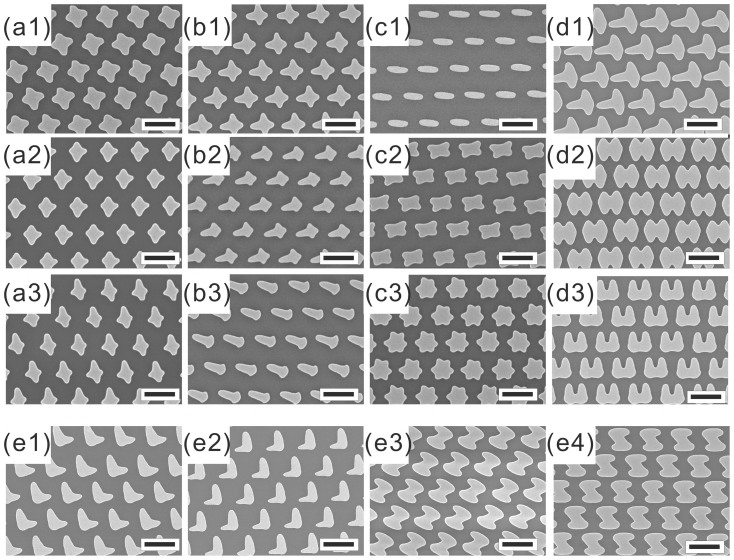
SEM images of the nanostructures fabricated. (a1) to (a3) all with E_1_(0 cm, 0°, 100 s) and varying exposure duration so E_2_(0 cm, 90°, 100 s), E_2_(0 cm, 90°, 80 s), E_2_(0 cm, 90°, 60 s), respectively. (b1) to (b3) are results after E_1_(0 cm, 0°, 100 s) and varying the shift distance (S_x_) so E_2_(3 cm, 90°, 100 s), E_2_(6 cm, 90°, 100 s), E_2_(7.5 cm, 90°, 100 s), respectively. (c1) to (c3) are results after single, double, and triple exposures by rotating the lamp 60° after each exposure. The exposure durations are all 100 s. (d1) E_1_(0 cm, 0°, 100 s)/E_2_(7.5 cm, 90°, 360 s) (d2) E_1_(0 cm, 0°, 100 s)/E_2_(7.5 cm, 90°, 300 s)/E_3_(7.5 cm, −90°, 360 s) (d3) E_1_(6 cm, 0°, 300 s)/E_2_(6 cm, 90°, 300 s)/E_3_(6 cm, 180°, 300 s) (e1) to (e4) are possible planar chiral metamaterials that can be fabricated. (e1) and (e2) E_1_(0 cm, 0°, 150 s)/E_2_(7.5 cm, ±90°, 300 s). (e3) and (e4) E_1_(0 cm, ±45°, 100 s)/E_2_(7 cm, 90°, 300 s)/E_3_(7 cm, −90°, 300 s). The Au thickness of all the nanostructures is 15 nm and the scale bar indicates 2 μm.

Fig. 6 illustrates the scanning electron microscopy (SEM) images of different shapes of structures that can be fabricated using the proposed method. Fig. 6(a1) demonstrates a cross array fabricated by performing the 2^nd^ UV exposure after rotating the lamp for 90°. The durations for both exposures are 100 s and the resulting cross structures exhibit two legs with similar length. The length for each leg can be separately controlled by changing the duration for each UV exposure. Figs. 6(a2) and (a3) demonstrate two un-even cross arrays which exhibit two different leg lengths. In both figures, the 1^st^ exposure for both samples are kept the same at 100 s and the 2^nd^ exposure is reduced to 80 s and 60 s, respectively. It is possible to fabricate a cross structure with an offset center position. This can be simply done by changing the sample position and then performing the 2^nd^ exposure. The durations for the first and second exposures for all the samples are set as 100 s. Figs. 6(b1) to (b2) illustrated the SEM images of the resulting cross arrays. The shift of the second leg is clearly observed in each image and the shift becomes larger when S_x_ becomes larger. The shift is about 140 nm when S_x_ is 3 cm and increases to 450 nm when S_x_ is 7.5 cm. It should also be noted that the length of the second leg becomes shorter as the shift of sample location becomes larger, which is reasonable since the sample is further away from the lamp.

It is also possible to fabricate more complicated nanostructures by changing the angle between exposures and the amount of exposure steps taken. Fig. 6(c1) illustrates the SEM image of the nano-ellipse array after a single exposure for 100 s. When performing the 2^nd^ exposure, the sample is clock wisely rotated by only 60°, results in a butterfly array shown in Fig. 6(c2). By rotating the sample again by another 60°, the star array shown in Fig. 6(c3) is fabricated. The duration of the 2^nd^ and 3^rd^ exposures are both kept at 100 s. It is also possible to perform the multiple exposures after shifting the sample location and rotating the sample. In an attempt to preciously describe the exposure parameters, a notation is introduced. For each exposure, a set of parameters are combined in the following manner E_X_(Sx, Angle, Duration), where “X” indicates the exposure number, “S_x_” the shift distance, “Angle” the angle between the sample and the lamp, and “Duration” the exposure duration. For example, to fabricate a T-shaped element arrays shown in Fig. 6(d1). The exposure sequence is E_1_(0 cm, 0°, 100 s)/E_2_(7.5 cm, 90°, 360 s). The H-shaped elements as shown in Fig. 6(d2) can be fabricated via the following exposure sequence: E_1_(0 cm, 0°, 100 s)/E_2_(7.5 cm, 90°, 300 s)/E_3_(7.5 cm, −90°, 300 s). To fabricate U-shaped element arrays shown in Fig. 6(d3), we can perform the following three exposures: E_1_(6 cm, 0°, 300 s)/E_2_(6 cm, 90°, 300 s)/E_3_(6 cm, −90°, 300 s).

It is also possible to fabricate PCMs simply by tweaking the exposure sequences, as shown in Figs. 6(e1)–(e4). PCMs consist “flat” chiral elements possessing no line of symmetry in the plane. A planar object is said to be chiral if it cannot be brought to congruence with its mirror image unless it is lifted from the plane^18^. For example, the L-shaped element array shown in Fig. 6(e1) is fabricated after double exposures: E_1_(7 cm, 0°, 300 s)/E_2_(7 cm, 90°, 150 s). If the sample is rotated for −90° and the same 2^nd^ exposure is performed, an inverse L-shaped element array appears as shown in Fig. 6(e2). Z-shaped element array is also a type of PCMs as shown in Fig. 6(e3). It can be fabricated by performing the triple exposures: E_1_(0 cm, 45°, 100 s)/E_2_(7 cm, 90°, 300 s)/E_3_(7 cm, −90°, 300 s). The mirror image of Z as shown in Figure 6(e4), can be fabricated simply by changing the 1^st^ exposure angle from 45° to −45°.

## Discussion

SEM images shown in Fig. 6 have clearly demonstrated the uniqueness of this method. In other method such as nano-imprint or nano-stencil lithography, it is almost impossible to make such complicated structures by doing the second and third processes due to the difficulty in re-alignment. Usually, a new hard mold is necessary. Any modification, such as the lengths for each leg or the angle between the legs, also requires new mold fabrication. In addition, both nano-imprint or nano-stencil lithography require to use low throughput nanofabrication method to fabricate the necessary hard mold. It is not practical to fabricate such a hard mold that covers an area as large as 1 cm^2^ due to the extra long fabrication time. Therefore, a more expensive micro-FTIR instrument is usually required to detect IR signal from the fabricated nanostructures. The focusing of the IR beam is also a potential problem if the IR responses from nanostructures are sensitive to the incident angle of IR beam. The fabricated ellipse array using the proposed method can easily cover an area as large as 1 cm^2^ and is big enough for conventional FTIR instruments, resulting easier and more accurate measurement. In addition, the fabricated ellipse arrays are uniform in size and cover a large area. Ellipse array with similar optical properties can be repeatedly fabricated. These advantages will make this proposed method as an important tool for future industrial applications.

These fabricated structures are very similar to the reported structures that can be applied in various applications. For examples, the un-even cross array demonstrated in Figs. 6(b1) to (b3) is reported to be a key component for dual-band IR PAs^12^. The H-shaped MMs demonstrated in Fig. 6(d2) were reported to be used for EIT applications^13^,^14^. The U-shaped MMs demonstrated in Fig. 6(d3) was used as Fano-resonant asymmetric metamaterials to detect protein monolayer^16^. Recently, the L-shaped MMs were also demonstrated to enhance the second harmonic generation (SHG) of light^54^,^55^. It is possible to fabricate more complex MMs by performing more than three exposures or depositing multiple layers. We believe the proposed method is powerful enough to fabricate most IR MMs and fabricate them economically in a large scale. It should be noted that each of the above MMs and PCMs exhibits interesting optical properties and applications in various research topics. It is not practical to discuss all of them in detail in this research article. Furthermore, the fabrication throughput of this proposed method can be improved by employing the concepts from micro-lens (MLA) array lithography, which removes the contact problem between the substrate and the spheres^56^,^57^.

In conclusion, we have demonstrated the fabrication of nano-ellipse arrays simply using NLL by replacing the light source with a regular UV lamp. The light is propagating differently along the directions parallel and perpendicular to the lamp directions due to the existing of the back reflector. The orientation of the nano-ellipse can be precisely controlled since the long axis is found to be parallel to the lamp direction. We also found out that the aspect ratio of the nano-ellipses strongly depends on the diameters of nanospheres. The larger the nanosphere, the ratio becomes larger. Nano-ellipse arrays with the length of major axis between 200 nm to 1.4 μm are successfully demonstrated, with their LSPR response between visible to mid-infrared. It is also demonstrated that tuning the periodicity and exposure time can shift the LSPR response to an optimized spectra range for SEIRA. We also demonstrated a methodical ways to design and fabricate various complicated MMs or PCMs simply by precisely controlling the multiple exposures. The versatile and low-cost nanofabrication method, which can also perform high-throughput nanofabrication, should pave the way for more industrial applications of IR MMs or PCMs.

## Methods

Highly resistive silicon substrates that were cut into size of 2 × 2 cm^2^ were used in this study. After ultrasonically cleaned in isopropyl alcohol, the substrates were rinsed with deionized water and blow-dried by nitrogen. The nanospheres used in this study were polystyrene (PS) spheres with various diameters (Polyscience Inc.). In the beginning, a thin layer of PR was spin-coated on top of the substrate followed by soft-baked at 100 °C for 3 minutes. The estimated thickness of the PR was about 500 nm. A single-layered hexagonal and close-packed micro-/nano-sphere array was subsequently formed via convective self-assembly method^58^. The sphere array serves as a photolithography phase mask for the subsequent exposure of UV light (λ = 365 nm) using a commercial UV lamp (Spectroline ENF-280/FE). The exposed-PR thin film was developed and the remaining spheres were removed. Au thin films are thermally evaporated onto the samples and nano-ellipse arrays revealed after the lift-off process. The solvent used for lift-off is acetone. A scanning electron microscope (SEM; JEOL JSM-6340F field emission scanning electron microscope) was used to analyze the corresponding nanostructures after each step. The lengths of major and minor axes of the fabricated nano-ellipses are subsequently analyzed from the SEM images. The UV light intensity is measured by commercial power meter (Ophir NOVA/PD-300UV). Optical absorption spectra of the fabricated arrays were measured using a commercial Fourier-transform Infrared (FTIR) spectrometer (Bruker VERTEX 70) purged with nitrogen gas and equipped with a KRS-5 variable polarizer from Pike, a Deuterated-triglycine-sulfate (DTGS) detector at a spectral resolution of 4 cm^−1^. Electromagnetic simulations were performed with the three-dimensional finite-difference time-domain (3D-FDTD) method, using a freely available software package^59^. The plasma frequency and damping constant of the Au are set at 1.32 × 10^16^ Hz and 0.68 × 10^14^ Hz, respectively.

## Author Contributions

Y.C.C. conceived the design, supervised the whole projects, performed the electromagnetic simulations and wrote the manuscript. S.C.L., H.C.C., S.M.W. fabricated the samples and performed various characterizations. T.D.T. and T.F.G. performed the FTIR measurements. All authors reviewed the manuscript.
